# Research progress in heterogeneity of dental mesenchymal stem cells

**DOI:** 10.1038/s41368-026-00433-8

**Published:** 2026-04-03

**Authors:** Hanqi Fu, Peng Chen, Zuping Wu, Xiangwei Kong, Li Xu, Xinyi Fang, Chi Liao, Xinlei Yu, Qianming Chen, Xiaoyan Chen

**Affiliations:** 1 Stomatology Hospital, School of Stomatology, Zhejiang University School of Medicine, Zhejiang Provincial Clinical Research Center for Oral Diseases, Key Laboratory of Oral Biomedical Research of Zhejiang Province, Cancer Center of Zhejiang University, Engineering Research Center of Oral Biomaterials and Devices of Zhejiang Province, Hangzhou, China; 2 Department of Stomatology, Jinling Hospital, Medical School of Nanjing University, Nanjing, China

**Keywords:** Cell biology, Stem cells

## Abstract

Dental tissues development involves two distinct cell lineages: mesenchymal cells (derived from the cranial neural crest) and epithelial cells (derived from oral ectoderm and pharyngeal epithelium). Emerging evidence highlights the remarkable functional heterogeneity of cranial neural crest-derived dental mesenchymal stem cells (DMSCs), exhibiting pluripotency, self-renewal, and differentiation capacities. This heterogeneity enables a single DMSC population to generate specialized subpopulations with unique roles in teeth and periodontal tissues formation. Significant progress has been made in characterizing six major types of DMSCs and two populations of closely related cells: Tooth germ progenitor cells (TGPCs) and dental follicle stem cells (DFSCs), critical during early morphogenesis; Stem cells from human exfoliated deciduous teeth (SHEDs) and apical papilla stem cells (SCAPs), pivotal for root development; Dental pulp stem cells (DPSCs), periodontal ligament stem cells (PDLSCs), gingival mesenchymal stem cells (GMSCs) and alveolar bone mesenchymal stem cells (ABMSCs), essential for maintaining and regenerating mature dental tissues. A key breakthrough has unveiled the development and hierarchy of DMSCs by applying new techniques like single-cell RNA sequencing (scRNA-seq). To integrate insights into the development of teeth and periodontal tissues, this review synthesizes current knowledge on both developmental heterogeneity and subpopulation heterogeneity within DMSCs and related cells. These insights not only advance fundamental understanding of the developmental mechanisms of teeth and periodontal tissues, but also establish a promising framework for achieving more efficient tissue regeneration and repair engineering.

## Introduction

**Fig. 1:**
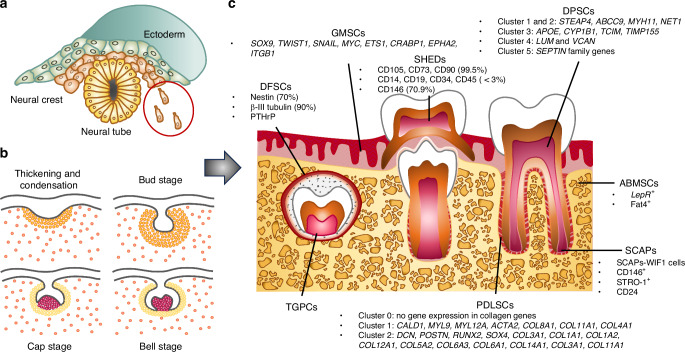
Schematic representation of odontogenesis and lineages of DMSCs and related cells. **a** Cranial neural crest-derived mesenchyme constitutes the primary source of odontogenesis. **b** These cells progress through epithelial thickening, invagination and subsequent developmental stages (the bud-cap-bell stages), ultimately forming dental and periodontal tissues. **c** Single-cell transcriptomics reveals distinct expression profiles corresponding to diverse differentiation trajectories. TGPCs tooth germ progenitor cells, DPSCs dental pulp stem cells, DFSCs dental follicle stem cells, PDLSCs periodontal ligament stem cells, SHEDs stem cells from human exfoliated deciduous teeth, GMSCs gingival mesenchymal stem cells, SCAPs apical papilla stem cells, ABMSCs alveolar bone mesenchymal stem cells

The formation of teeth and periodontal tissues constitutes a dynamically integrated system, which contains multiple components, including hard tissues such as enamel, dentin, cementum, and alveolar bone, as well as soft tissues like dental pulp and periodontal ligament (PDL). These sophisticated structures collectively originate from precisely regulated embryonic developmental processes. Initial epithelial thickening creates the dental lamina, followed by mesenchymal invagination and condensation, and ultimately progressing through the morphogenetic bud-cap-bell stages that determine the tooth shape (Fig. 1a, b).^1^ Each stage is well-organized, relying on spatiotemporally specific signaling pathways and transcriptional factors to achieve coordinated control and ultimately the formation and maturation of teeth and periodontal tissues.^2^ From the cellular lineage perspective, this developmental process is closely associated with a specialized population of stem cells—dental mesenchymal stem cells (DMSCs). Major types include dental follicle stem cells (DFSCs), stem cells from human exfoliated deciduous teeth (SHEDs), apical papilla stem cells (SCAPs), dental pulp stem cells (DPSCs), periodontal ligament stem cells (PDLSCs), and gingival mesenchymal stem cells (GMSCs).^3–5^ Given the integrity from development to tissue formation, two additional cell populations have been included in this review due to their strong correlation with DMSCs. These are tooth germ progenitor cells (TGPCs), which serve as precursor cells for some DMSCs, and alveolar bone mesenchymal stem cells (ABMSCs), which are region-specific mesenchymal stem cells (MSCs) (Fig. 1c).^3–5^ These cells all originate from multipotent cranial neural crest cells (CNCCs). Through epigenetic modifications (including DNA methylation, histone modifications, etc.) and lineage-specific transcriptional programs,^6^ these neural crest-derived MSCs finally generate the entire teeth and periodontal structures except enamel, which is formed by oral ectoderm-derived epithelial stem cells.^7^

Emerging evidence has shown that DMSCs, despite sharing the same origin, exhibit significant heterogeneity in molecular features, cell fate, and functional output.^8,9^ On the one hand, DMSCs exhibit developmental heterogeneity. TGPCs, DFSCs, SHEDs, and SCAPs emerge transiently during development and execute stage-specific programs through spatiotemporal differentiation. When the initial TGPCs are modulated by signals from adjacent tissues and the in vivo microenvironment,^7^ they can diverge into distinct differentiation trajectories even though from the same progenitor pool, as exemplified by DFSCs, which significantly contribute to periodontal tissue formation.^10^ On the other hand, other DMSCs (including DPSCs, PDLSCs, and GMSCs) and ABMSCs residing in different adult tissues demonstrate subpopulation heterogeneity. For instance, DPSCs, although constituting a minor fraction (about 5%) of the total dental pulp cellular population,^11^ are indispensable for the development, differentiation, and regeneration of various pulpal cell lineages. Previous investigations have highlighted that distinct anatomical niches may serve as possible reasons for functional discrepancies between highly similar stem cells localized in different dental chambers.^12,13^ Upon exposure to exogenous injurious stimuli, the self-repair and regenerative potential of teeth and periodontal tissues may be mediated through the activation of certain DMSCs. Hence, inherent heterogeneity of DMSCs not only constitutes a developmental basis for constructing complex tissue architectures, but also represents the cellular reservoir enabling lifelong self-repair and regenerative capacities in adult tissues.

This review examines the heterogeneity of DMSCs and homologous cells closely related to DMSCs through two dimensions: the developmental and subpopulation heterogeneity (the tissue origins, developmental stages, and key heterogeneity of the 8 main cells populations are summarized in Supplementary Table 1). Firstly, we analyze the stage-specific developmental heterogeneity during tooth germ formation and morphogenesis by their deterministic influence on subsequent tissue patterning. Such spatiotemporal specificity in developmental mechanisms could lay the molecular foundation for biomimetic regeneration strategies. Secondly, we try to conduct an in-depth analysis of subpopulation profiles within adult DMSCs and ABMSCs, particularly identifying functionally distinct subpopulations which may refine tissue engineering protocols. Advanced techniques like single-cell RNA sequencing (scRNA-seq)^14,15^ are used to bridge basic research with clinical translation, offering opportunities for functional validation and therapeutic innovation. The integration of technological innovation and mechanism exploration opens new avenues for targeting the regulatory networks underlying heterogeneity and precision regenerative therapies.

## Development of teeth and periodontal tissues

The development of teeth and periodontal tissues starts from the migration and proliferation of CNCCs, which are derived from the lateral neural plate crest at the third week of embryonic development and then initiate a directional migration program (Fig. 1a).^7,16^ During the fourth week, CNCCs undergo extensive ventral migration, establishing the primitive branchial arch structures.^7,16^ By the sixth week, tooth morphogenesis begins, and at this time, two different embryonic lineages can be distinguished: One is the cranial neural crest-derived mesenchymal cells, which give rise to dental pulp, dentin, alveolar bone, and PDL in the future; The other is oropharyngeal ectoderm-derived epithelial cells that produce enamel-secreting ameloblasts and their supporting cells.^7^ At this moment, following the interaction between ectoderm and neural crest mesenchyme, the primary oral epithelium undergoes localized thickening and subsequently invaginates downward into the underlying mesenchyme to form the tooth germ.^2,7,17^ The research on the developmental biology shows that the tooth germ is composed of three functional units: Enamel organ, dental papilla, and dental follicle (Fig. 1b). The enamel organ, which originates from ectoderm, is responsible for enamel formation through matrix secretion and mineralization.^18^ Besides ameloblasts, non-ameloblast cell populations play critical regulatory roles during development although their functional roles remain poorly characterized. However, these cells vanish in the mature enamel, resulting in the inability of enamel to spontaneously repair itself after damage.^19^ Therefore, biologically induced enamel regeneration faces great challenges.^20^ Several types of DMSCs have been found in the dental papilla and dental follicle tissues, exhibiting remarkable plasticity. They undergo dynamic phenotypic changes and fate decisions under the influence of in vivo microenvironment, following different stage-specific differentiation trajectories.

According to the morphological transformation characteristics at different developmental stages, the tooth germ goes through three precisely orchestrated sequences: The bud-cap-bell stages. This continuous process involves complex epithelial-mesenchymal transformation (EMT), and it depends on a variety of cytokines and signaling pathways, such as bone morphogenetic protein (BMP), fibroblast growth factor (FGF), the Wnt signaling pathway, and the Shh signaling pathway, etc.^1,2,21^ There are also transcription factors (for example, *Foxp4* is responsible for the formation of PDL, while *Prrx1* is responsible for molar morphogenesis), cis-regulatory elements and epigenetic modifiers, etc.^6,22^ These factors and pathways construct a sophisticated gene regulatory network (GRN), which is responsible for precisely regulating the proliferation, differentiation, and migration patterns of DMSCs.^1,6,21,22^ In terms of precise control at the cellular level, it has been found through scRNA-seq and spatial genomic analysis of the mouse development model that CNCCs initially migrate to the oral region of the first pharyngeal arch.^6,22^ On embryonic day 12.5 (E12.5), a population of progenitor cells with the dual differentiation potential of osteogenesis and dentinogenesis appears.^6,22^ These cells maintain the characteristics of homogeneity and pluripotency until E13.5.^6,22^ Then during the period from E14.5 to postnatal day 3.5 (P3.5), these cells gradually differentiate into two lineages: The odontoblast lineage forming the dental pulp and dentin, as well as the dental follicle cell lineage forming the periodontal tissues.^6,22^ In recent studies on human fetal development, researches have shown that long non-coding RNAs (lncRNAs) may participate in the EMT through cis-regulatory effects on neighboring genes, such as *LINC00511* (associated with enamel formation) and *DLX6-AS1* (linked to dentin formation).^23^ In addition, in vitro single-cell transcriptome analysis has discovered a subpopulation of *Alx3*^*+*^*/Barx1*^*+*^ DMSCs, which can differentiate into an odontogenic cluster characterized by the upregulation of *Pax9/Bmp3* and *Lhx6/Dmp1* expression.^24^ This multilayered regulatory framework, encompassing diverse functional factors, ensures the spatiotemporal characteristics of DMSCs in proliferation, migration, and histodifferentiation throughout teeth and periodontal tissue development.

Although the developmental process of teeth and periodontal tissues has been basically clarified, the spatiotemporal dynamics at cellular lineage resolution remain incompletely mapped. Summarizing the functions and roles of various DMSCs and the closely related cells during the developmental process will fill this gap. This section artificially partitions the developmental timeline according to key morphogenetic milestones, and summarizes the cell populations that play a major role in each period. Firstly, TGPCs arise in the initial tooth germ formation as heterogeneous progenitors for the entire teeth and periodontal complex. Following the formation of dental follicle, DFSCs transition into lineage-committed progenitors, which orchestrate periodontal tissues assembly during the critical developmental transition phase. In terminal phase of tooth root morphogenesis, SCAPs are identified in the apical area of immature teeth, serving as architects of tooth root formation and showing surprising neural differentiation potential. Each type of these cells, with their unique characteristics, differentiation potential, and regulatory pathways, holds the key to unlock a deeper understanding of how teeth and periodontal tissues form, grow, and regenerate.

### (1) TGPCs are the earliest DMSCs-related cells that emerge during development

Tooth germ cells undergo complex differentiation processes to yield multiple cell lineages—ameloblasts, odontoblasts, dental pulp cells, and PDL cells—that orchestrate teeth and periodontal tissues formation. Initial investigations identified that cells from human third molar tooth germs exhibited high proliferative capacity and multilineage differentiation potential towards osteoblasts, adipocytes, and neurogenic cells under specific in vitro conditions.^25^ These cells, then designated as TGPCs, have been primarily studied in mature tissues through in vitro experiments.^25^ And they actually resemble an assemblage of progenitor cell populations in early developmental stages. However, in vivo odontogenesis is a continuous developmental process, during which TGPCs exist transiently in early stages and undergo multidirectional differentiation subsequently. Research highlights the critical role of TGPCs in osteogenesis and dentinogenesis in the bell stage,^26^ which is recognized as a pivotal phase in tooth development. This process is mediated by molecular regulators, including ALK5 (a kinase regulating tooth initiation and early mandibular patterning through transforming growth factor beta-receptor (TGF-βR2)-independent pathway) and TGF-β3 (a TGF-β signal activator). Following dentinogenesis, the encapsulated dental papilla differentiates into dental pulp tissues. Within mature dental pulp, a population of cells exhibiting self-renewal capacity and multilineage differentiation potential is designated as DPSCs. There may be a developmental relationship between TGPCs and DPSCs. Combining scRNA-seq and spatial transcriptome (ST) techniques, it was discovered that dental papilla cells evolved into two distinct cellular domains, with the increasing proportion of coronal dental papilla cell clusters (*FGF3*^+^, *TFAP2A*^+^, and *CPVL*^+^).^27^ Notably, the *FGF3*^+^ cell population was considered a precursor of odontoblasts.^27^ Overall, TGPCs may represent a potentially high-quality cell source for regenerative medicine due to their closer proximity to the developmental origin. However, there is currently insufficient robust and comprehensive research evidence to substantiate this claim.

### (2) DFSCs, critical for periodontal tissue formation, emerge at the cap stage

During the cap stage, ectomesenchymal cells surrounding the enamel organ and dental papilla edge tightly integrate into the connective tissue layer and then form the dental follicle. This essential structure is responsible for the development of periodontal tissues. DFSCs existing at this stage not only regulate the eruption of teeth, but also contribute to the formation of alveolar bone. These cells demonstrate remarkable plasticity. They could differentiate into cementoblasts, fibroblasts, and osteoblasts under the guidance of interconnected signaling pathways, including TGF-β, Hippo, and Wnt, which are modulated through the interaction of BMP2/BMP4 and their pseudoreceptor BAMBI.^21,28–30^ The initial isolation of DFSCs from the dental follicle of human third molars revealed their unique molecular signature, prominently featuring the expression of NOTCH1—a transmembrane receptor critical for cell fate determination—along with neural markers such as nestin and β-III tubulin.^31,32^ NOTCH1 activation in DFSCs enhances proliferative capacity by accelerating G1/S phase transition and at the same time suppresses premature differentiation, a mechanism that underscores their self-renewal potential.^32^ Approximately 70% of DFSCs display the expression of nestin (a marker of neural progenitor cells), while about 90% express β-III tubulin (an early neuronal marker).^32^ Intriguingly, the co-expression of neural progenitor markers and early neuronal markers suggests retained neural crest lineage features, hinting at their capacity for neurogenic differentiation.

Both in vitro and in vivo studies have highlighted the importance of the *Runx2/Nell-1* axis in the osteogenic differentiation of DFSCs. *Runx2* drives transcriptional activation by directly binding to the osteoblast-specific element (OSE) in the NELL-1 promoter, which is crucial for bone formation and craniomaxillofacial development.^29^ This indicates that this signaling axis is essential for regulating the osteogenic differentiation of DFSCs. Furthermore, lineage-tracing studies have identified a distinct DFSCs subpopulation, which expresses parathyroid hormone-related protein (PTHrP) and regulates periodontal attachment formation through autocrine parathyroid hormone (PTH)/PTHrP receptor signaling.^15,29,30^ And scRNA-seq further reveals that PTHrP^+^ DFSCs exhibit periodontal hard tissue differentiation potential (e.g., osteogenic and cementogenic ability), as PTHrP-mediated signaling serves as a definitive marker for osteogenic differentiation and a key regulatory molecule for cementogenesis.^33,34^ Additionally, a stem cell subpopulation persists in mature periodontal tissues, which could respond to periodontal injuries through paracrine PTH/PTHrP signaling and regulate occlusal forces by the canonical Wnt pathway.^33^ From a developmental biology perspective, it is hypothesized that PDLSCs in adult tissues may derive from undifferentiated DFSCs which reside in periodontal tissues during tissue formation and maturation.^35^ This means DFSCs may serve as the primary cellular source for periodontal tissues. Collectively, these findings elucidate the characteristic subpopulations of DFSCs and their multi-layered signaling regulatory networks, which advance our understanding of the periodontal tissue development mechanism.

### (3) SCAPs are a specialized type of DMSCs that emerge during tooth root development

When the development of the dental crown is about to complete, reaching the cap stage and the bell stage, the proliferation and differentiation of progenitor cells within the cervical loop ensure further elongation and invagination of the dental epithelium.^1,33^ This step simultaneously initiates the tooth root formation and influences the differentiation of odontoblasts from undifferentiated mesenchymal cells and cementoblasts from follicle mesenchyme.^36^ The apically extending bilateral epithelial walls (an amalgamation of the internal and external enamel epithelium) result in the formation of Hertwig epithelial root sheath (HERS).^36^ HERS is instrumental in defining the root morphology and facilitating cementum formation through EMT.^36,37^ In incompletely developed tooth phase, DMSCs, especially those found in the immature apical papilla tissues, exhibit distinctive characteristics. The apical papilla refers to the soft tissues that are loosely attached to the apices of immature permanent teeth and can be easily detached with a pair of tweezers.^38^ Studies have already isolated a distinctive type of DMSCs from the root apex of young teeth, which were designated as SCAPs.^39^ In particular, SCAPs can only be isolated at the tooth root development stage of tooth maturation as these cells subsequently evolve into the corresponding structures during the formation of tooth crown and root.^38^

Current research on the identification and understanding of SCAPs are relatively limited, while some surface markers may help us find the characteristics of SCAPs. For example, CD24, a vastly glycosylated cell protein, not only maintains SCAPs in an undifferentiated state but also regulates their adipogenic potential through unresolved signaling mechanisms.^40^ Some studies^41,42^ even view CD24 as a specific marker of SCAPs, as it is undetectable in other DMSCs, including DPSCs.^41^ The expression of CD24 may suggest the stemness state of SCAPs.^42^ Once passing to the 10^th^ generation, its expression will decrease to nearly 0%, which can be explained as cells have begun to leave the undifferentiated state and transition into the osteoblast lineage.^42^ Moreover, proteomic analysis revealed that SCAPs exhibiting high CD44 expression mediate the interactions between apical tissues and enamel organs, thereby promote the differentiation of dental pulp and dentin cells.^43^ Additionally, in vitro experiments demonstrate that SCAPs can produce a wide range of cytokines, such as insulin-like growth factor-1 (IGF-1), IGF binding protein-6 (IGFBP-6), interleukin-10 (IL-10) and TGF-β3.^44^ These cytokines significantly influence the biological behavior of surrounding tissues and cells.^44^ Another study^45^ has explored the function of Wnt-1 inducible factor 1 (WIF1, a member belonging to the secretion regulator family of Wnt proteins) in the dentin differentiation of SCAPs.^45^ Results have indicated that WIF1 enhances alkaline phosphatase (ALP) activity and in vitro mineralization by activating the transcription factor Osterix (OSX), thereby strengthening the dentin differentiation of SCAPs.^45^ This might imply that SCAPs-WIF1 cells possess stronger odontogenic potential.

ScRNA-seq and GRN analyses have unveiled remarkable heterogeneity among SCAPs, which distinct subpopulations exhibit specialized functions. In terms of differentiation trends, one subpopulation expressing *DLX5*, a gene associated with tooth formation, demonstrates heightened odontogenic potential. Another subpopulation, enriched for *DIO2* expression, exhibits strong differentiation capacity and osteogenic activity under mineralization conditions.^46^ Furthermore, another CD146 expressing subpopulation may be linked to angiogenesis and endothelial cell activity, while the STRO-1^+^ subpopulation contains neurogenic features, making it a promising candidate for stem cell-based therapies in central and peripheral nerve injury.^38,42^ Compared with other subpopulations, double-positive (both STRO-1^+^ and CD146^+^) subpopulation presents higher proliferation rate and odontogenic differentiation potential, and also expresses key embryonic markers.^38,42^ These findings highlight the potential of SCAPs as a source of neural stem cells (NSCs). Later, research has already confirmed the potency of in vitro-derived NSCs from SCAPs, which is expected to be used for exogenous transplantation in stem cell-based therapies for neurodegenerative diseases.^47^ These insights into SCAPs—including developmental regulation, molecular diversity, and therapeutic potency—underscore their unique position at the intersection of developmental biology and regenerative medicine.

A systematic analysis of odontogenesis underscores the indispensable roles of temporally emerging progenitor stem cell populations, each contributing unique functional modules to tooth development (specific features are also summarized in Supplementary Table 2). Though sharing the same neural crest origin, these cells in fact emerge sequentially, occupy specialized niches and adopt divergent differentiation trajectories. Among them, TGPCs just exist briefly in early developmental stages and show heterogeneity during the whole progress. DFSCs and SCAPs also demonstrate heterogeneity, which have been identified different characteristic subpopulations showing different function tendency. This developmental sequence reveals a fundamental biological strategy: DMSCs are pre-patterned into fate-committed effector subpopulations guiding tissue-specific differentiation and undifferentiated reserve pools maintaining regenerative capacity. It provides critical insights into the self-organization of mineralized tissues but simultaneously poses significant clinical challenges. The absence of these progenitors in adult tissues—particularly TGPCs and SCAPs, with their narrow developmental windows—severely limits their isolation and functional characterization.

Consequently, research focus has pivoted predominantly toward DMSCs derived from mature teeth and periodontal tissues, such as DPSCs and PDLSCs. Recent advancements highlight their potential in regenerating dentin-pulp complexes and periodontal apparatus. Meanwhile, new emerging technologies facilitate biomaterial-guided differentiation to enhance lineage specificity, as well as genome editing to modulate regenerative pathways. Although their inherent multipotency enables diverse therapeutic applications, uncontrolled heterogeneity compromises clinical reproducibility. For instance, DPSCs may concurrently contain odontoblast-committed, neurogenic, and angiogenic subpopulations, requiring refined isolation protocols to ensure therapeutic consistency. Therefore, it’s necessary to conclude the research progresses in the heterogeneity of these adult DMSCs.

## DMSCs in adult tissues and their subpopulation heterogeneity

Following the maturation of teeth and periodontal tissues, DMSCs persist as highly heterogeneous populations within specialized niches, retaining their developmental plasticity. Originating from the lateral neural plate crest during craniofacial morphogenesis,^48^ these cells inherit the remarkable adaptability of their neural crest progenitors, whose fate decisions are dynamically regulated by microenvironmental cues.^49,50^ Correspondingly, the niche comprises a complex milieu of growth factors, extracellular matrix (ECM) components, and biophysical signals that collectively modulate DMSCs’ behavior to balance self-renewal with lineage-specific differentiation.^12,13,51,52^ For instance, DPSCs residing in a specialized niche—the vascular nerve bundle of dental pulp—engage in intimate crosstalk with perivascular endothelial cells.^53^ This interaction is essential for maintaining the DPSCs pool and sustaining their functionality.^54,55^ Despite their therapeutic promise, in vivo study of DMSCs remains challenging due to the dynamic nature of their niches, the technical limitations in isolating pure populations, and the transient activity of key signaling pathways. Consequently, research has prioritized DMSCs derived from accessible adult tissues, such as DPSCs and PDLSCs, to elucidate their roles in tissue repair and regeneration. Notably, these populations exhibit marked functional heterogeneity, evidenced by divergent differentiation potentials, variable marker expressions, and distinct secretory profiles. Advancements in single-cell transcriptomics, spatial omics, and lineage-tracing technologies are now critical for dissecting subpopulation heterogeneity. Such tools enable the identification of rare subpopulations, such as quiescent “reserve” stem cells versus actively differentiating progenitors, and the clarification of their roles in homeostasis versus repair.

### (1) Dental pulp stem cells (DPSCs)

**Fig. 2:**
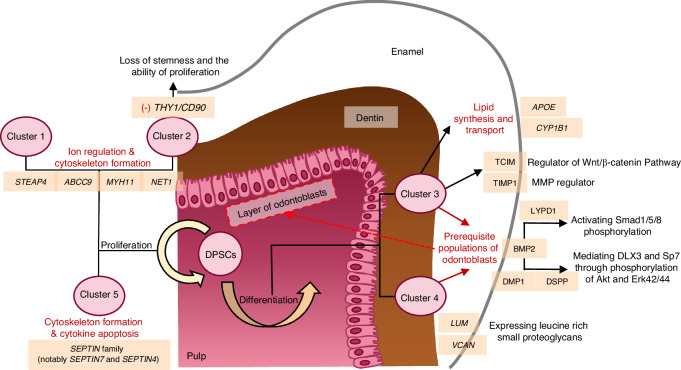
Atlas of DPSCs from Human Dental Pulp. Research has constructed the subpopulation feature of DPSCs *via* scRNA-seq and identified 5 clusters with different potentials

DPSCs were clonogenic and proliferative populations isolated from adult human dental pulp, and exhibited profound heterogeneity for diverse functional capabilities.^56^ Transcriptomic analyses have revealed distinct DPSCs subpopulations characterized by differential expression of *MYH11* (smooth muscle myosin heavy chain 11), *THY1* (CD90) and *CCL2* (monocyte chemotactic factor).^57,58^ Furthermore, it has been found that there are osteogenic and odontogenic clusters expressing *DCN*, *COL1A1*, *COL1A2*, *FN1*, and *VCAN*, and neurogenic clusters expressing *S100A4*, *NEFM*, *NEFL*, *COL4A1*, and neurofilament assembly related gene sets.^57,58^ Several researchers have constructed the cell atlas^59^ of DPSCs from human dental pulp *via* scRNA-seq and five distinct cell clusters are defined (Fig. 2). Clusters refer to similar or identical types of cells but differ in functional orientation, which represents the multipotent heterogeneity of stem cells. Collectively, recent research has discovered the subsets with certain clear characteristics from the whole namely subpopulations, and deepened our understanding of the heterogeneity of DPSCs.

In terms of proliferation, certain subpopulations make important contributions to maintain the number of reserve stem cells. Cluster 1 and 2 specifically express genes relevant to ion regulation and cytoskeleton formation (e.g., *STEAP4*, *ABCC9*, *MYH11* and *NET1*).^59^ Cluster 1 is related to the function of histone acetyltransferase activity, while cluster 2 exhibits remarkably suppressed expression of *THY1/CD90.*^59^ Cluster 5 selectively expresses *SEPTIN* family genes, particularly *SEPTIN7* and *SEPTIN4*, which are cytoskeletal proteins potentially linked to proliferation or cytokine apoptosis.^59^ This cluster may represent the progenitor cell population of DPSCs with enhanced proliferation and differentiation potential.^59^ In vitro experiments involving the tissue repair molecule Nogo-A protein demonstrated that it maintained DPSCs’ stemness and inhibited pluripotent differentiation by increasing *THY1* levels.^60^ In vivo lineage tracking revealed that MSCs involved in repairing intervertebral disc injuries exhibited lower expression of NOTCH3 and THY1 during their transition to fibroblasts and loss of progenitor characteristics.^61^ The potency of proliferation and differentiation is vital for stem cell-based clinical therapeutic applications. Current studies have identified that the MCAM^+^ JAG1^+^ PDGFRA^−^ subpopulation of DPSCs can uphold the transcriptional characteristics of freshly isolated DPSCs in vitro, and demonstrates higher proliferation and trilineage differentiation potential.^62^ The vascular structure of dental pulp is crucial in both development and regeneration. Using scRNA-seq, researchers have identified subpopulations from vascularized cells in dental pulp. Among these, The PDGFRβ^+^ subpopulation with activated PI3K/AKT pathway exhibits high expression of fibronectin 1 (FN1), as well as strong proliferative activity and pro-angiogenic ability.^63^

Regarding differentiation, some subpopulations exhibit relatively clear differentiation directions. Key signaling pathways, including the RUNX2/NELL-1 axis, Sp1/noggin pathway, and classical Wnt/β-catenin pathway, are pivotal for hard tissues formation. Cluster 3 expresses *APOE* and *CYP1B1* involved in lipid synthesis and transport, as well as high expression of Wnt regulator TCIM and MMP regulator TIMP1.^59^ Moreover, CYP1B1, a member of the cytochrome P450 superfamily, could also induce mitochondrial dysfunction and excessive production of reactive oxygen species (ROS).^64^ The increased expression of CYP1B1 may indicate aging and function decreasing of stem cells.^64^ Cluster 4 distinctively expresses leucine-rich small proteoglycans (LUM and VCAN), resembling the odontoblast expression profiles. It is hypothesized that clusters 3 and 4 represent prerequisite MSCs populations for odontoblast differentiation.^59^ ScRNA-seq has identified that LYPD1 is exclusively expressed in pre-odontoblast clusters and regulates the BMP2-Smad1/5/8 signaling pathway to promote odontoblast differentiation.^65^ This makes LYPD1 a novel and critical marker for odontoblast differentiation.^65^ The BMP2 signaling pathway operates dentin and tooth formation through two mechanisms: a Smad-dependent pathway (activating Smad1/5/8 phosphorylation) and a non-Smad pathway (mediating DMP1 and DSPP expression *via* AKT and ERK 42/44 phosphorylation).^66^ Using Assay for Transposase-Accessible Chromatin with high-throughput sequencing (ATAC-seq) to analyze the induced odontogenic differentiation of DPSCs further revealed that cell chromatin accessibility undergoes selective remodeling, and the core mechanism is the construction of related enhancers, especially by constructing specific super enhancers at the *ALPL* locus (a key marker gene for tooth differentiation).^67^ The specific open chromatin peak contains Smad4 consensus elements, which can recruit differentiation related transcription factors such as Smad1/4.^67^ This has also been confirmed in mouse experiments, with temporal ATAC-seq showing that chromatin accessibility changes occur earlier than transcriptional upregulation of odontogenic specific genes (*Dmp1*, *Dspp*).^68^ However, non-Smad signaling through P38 and ERK kinases, as well as Wnt/β-catenin signaling in PITX2, inhibits odontoblast-related gene expression, thereby affects dental cells proliferation and differentiation.^69^

Scientists have categorized subpopulations into various osteogenic stages by osteoblast differentiation genes (*RUNX2*, *SP7, SATB2*, *HAND2*, *MEF2C*, *COL1A1*, and *ALP*).^70^ For example, a subpopulation of cells displaying abundant RUNX2 content and lower expression of SP7 represented MSCs in the early stage of osteogenic differentiation.^70^ From organizational perspective, the osteogenic ability of DPSCs is not the most necessary, but its differentiation potential has been proven to exist. RUNX2 regulates downstream gene *NELL-1* by binding to specific promoter elements, aided by transcriptional regulator Sp7/OSX, which maintains a balance in NELL-1 expression.^29^ NELL-1 enhances dentin formation and co-expresses with neuro-markers like GFAP, nestin, and β-III tubulin, highlighting its role in neural differentiation.^71^

DPSCs exhibit multi-directional differentiation potential and clinical application advantages, such as availability, long shelf life and easy preservation. Under specific conditions, they can differentiate into odontoblasts, osteoblasts, dentin-pulp and neuron-like cells, making them valuable resources for regenerative applications.^72,73^ However, DPSCs are currently used in limited quantities, which restricts their potential for dental and medical applications. Single-cell analysis has shown that cultured dental pulp cells primarily originate from the CTGF and IGFBP5 mesenchymal subpopulations. Among them, IGFBP5 cells with progenitor characteristics may drive odontogenic differentiation, while monolayer cultures preserve cellular heterogeneity.^74^ The impact of senescence on DPSCs, particularly the CD51^+^/PDGFR-α^+^ subpopulation, has been studied using transgenic lineage tracking and molecular techniques.^75^ Findings indicate that senescence reduces self-renewal and osteogenic differentiation potential, with decreased CD51 expression but stable PDGFR-α levels.^75^ Further research is needed to mitigate the effects of senescence and enhance the therapeutic potential of DPSCs.

Interestingly, the potential of DPSCs varies under different stimuli. For example, stimulation by Porphyromonas gingivalis up-regulates THBS1 and PTGS2 expressions, which could resist infection and improve mineralization-related differentiation via TGF-β/SMAD, NF-κB, and MAPK/ERK signaling.^73^ While, Enterococcus faecalis stimulation promotes fibroblast-like differentiation by increased ACTA2 expression.^76^ These results might improve the understanding of the pathogenesis of pulpitis and periapical periodontitis. Besides, complement related mechanisms have also been found in DPSCs in terms of immune regulations. As we know, complement system is one of the key components of the innate immunity. Recent studies demonstrate complement receptors (C5a receptors and C5a like 2 receptors, abbreviated as C5aR and C5L2) could promote dentin repair through DMP-1 expression or promote nerve fiber growth through brain-derived neurotrophic factor (BDNF) secretion.^77,78^ C5aR have a positive control effect on BDNF and nerve growth factor (NGF),^78^ while silencing of C5L2 will promote BDNF production through the P38 α-mediated MAPK pathway.^77^ These underscore the potential of DPSCs in inflammatory conditions and nerve repair.

### (2) Periodontal ligament stem cells (PDLSCs)

**Fig. 3:**
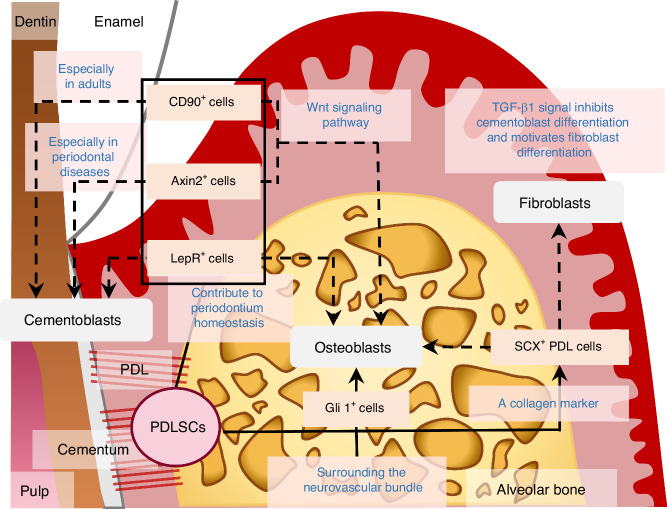
Atlas of PDLSCs from Human Periodontal Tissues. Research has identified several representative subpopulations of PDLSCs *via* techniques like scRNA-seq and figured out their potential respectively

Periodontal tissue is an important support for teeth, tightly connecting the cementum and alveolar bone. This structure contains stem cells with significant repair and regeneration capabilities, which are defined as PDLSCs through single-colony selection and magnetic-activated cell sorting.^79^ PDLSCs express MSC markers, including STRO-1 and CD146/MUC18, and exhibit multipotency with the ability to differentiate into fibroblasts, PDL progenitors and cementoblasts.^80^ Similar to DPSCs, PDLSCs could also be identified as 3 clusters.^57^ Previous studies have identified representative subpopulations, which may be categorized by expression features and so on (Fig. 3). Although this clustering method helps to distinguish subpopulations with diverse gene expressions, there are still some overlaps between them. For example, genes *COL8A1* in cluster 1 and *RUNX2* in cluster 2 both play a role in osteogenic differentiation of PDLSCs. Cluster 0, characterized by the absence of collagen gene expression, appears to represent precursor stem cells in their original state.^57^ In the following sections, we will explore the differentiation mechanisms and elaborate on the representative subpopulations in each cluster.

A study revealed that the leptin receptor^+^ (LepR^+^) PDLSCs gave rise to osteocytes and cementocytes in healthy and hard tissues regenerative conditions in LepR-cre and floxed-stopped reporter mice.^81^ However, the contribution of LepR/Tom^+^ cells to the lineage of hard tissue-forming cells was lower than the LepR/Tom^−^ subpopulation. Both LepR/Tom^+^ and LepR/Tom^−^ subpopulations showed self-renewal activity, which means LepR^+^ PDLSCs are a group of the heterogeneous populations.^81^ ScRNA-seq analysis has also identified LepR expression in human jawbone periosteal stem cells, which may be a distinct subpopulation of PDLSCs essential for periodontium homeostasis and bone regeneration during tooth socket healing.^82^ Additionally, recent research demonstrated that LepR^+^ cells play a vital role in osteogenesis by evaluating the bone remodeling induced by orthodontic force in a mouse model.^83^ Compressive and tensile stress caused the force-induced apoptosis of PDL cells, and then mobilized the activated LepR^+^ cells to the stressed area for bone formation.^83^ Furthermore, a basic helix-loop-helix transcription factor, SCX, expressing in mature PDL fibroblasts, has been identified as a marker of PDLSCs.^34^ The SCX^+^ PDLSCs appears to be a source of osteoblasts and other fibroblasts.^34^ Based on clustering characteristics, cluster 1 likely correlates with these subpopulations, as it expresses myofibroblast-related genes (*CALD1*, *MYL9*, *MYL12A* and *ACTA2*) and osteoblast-related genes (*COL8A1*, *COL11A1* and *COL4A1*).^57^ Prior studies also indicate that the Wnt signaling pathway significantly influences PDLSCs differentiation by promoting fibroblast differentiation when interacting with TGF-β1 and enhancing osteogenic differentiation.^52,84,85^ Gene mapping during PDLSCs osteogenic differentiation has revealed that the enzyme EZH2, involved in epigenetic regulation, stimulates differentiation through histone modification.^86^ This activation influences the expression of osteoblast-related genes, including *COL6A1* (a component of microfibrils), *VCAN* (a proteoglycan in the extracellular matrix), *CREB3L1* (a transcription factor involved in angiogenesis and osteoblast differentiation), and *RRBP1.*^86^

One of the key advantages of PDLSCs is their ability to differentiate into cementocytes in vivo, which could produce collagen fibers embedded in bone-like tissues—a process crucial for periodontal regeneration and repair. Cluster 2 is notable for expressing *DCN*, *POSTN*, *RUNX2*, *SOX4*, as well as *COL3A1*, *COL1A1*, *COL1A2*, *COL12A1*, *COL5A2*, *COL6A3*, *COL6A1*, *COL14A1*, *COL3A1*, *COL11A1.*^57^ Lineage tracking technology has discovered several special subpopulations, including Gli1^+^ PDLSCs.^81,87^ Gli1^+^ PDLSCs, negative for periodontal lineage differentiation markers such as OSX and COL1A1, are the main cell sources of alveolar bone development, and about 85% of bone cells in alveolar bone come from this subpopulation.^81,87^ Gli1^+^ cells surrounding the neurovascular bundle (NVB) in the apical zone have been linked to the origin of PDL component cells, with their differentiation positively regulated by the Wnt signaling pathway.^81^ Axin2^+^ cells are crucial for alveolar bone development, remodeling and regeneration. The αSMA lineage cells localized in the periapical region of PDL represent periodontal progenitors.^81,88^ These αSMA^+^ cells, without co-expression of COL1A1 (a mature osteoblast/osteocyte marker), could differentiate into fibroblasts, osteoblasts, cementoblasts, and cementocytes under both healthy and post-injury conditions.^81,88^ Interestingly, during root formation, both CD90^+^ and Axin2^+^ perivascular-associated cells give rise to cementoblasts, while in adult tissues, their source is limited to Axin2^+^ cells. In periodontal diseases, however, the origin of cementoblasts shifts to CD90^+^ cells.^81^

The unique structure of periodontal tissues lies in the close association between soft and hard tissues, which work together to bear periodontal forces and maintain tooth health. When periodontal tissues are damaged, PDLSCs might take on the responsibility, and the repair procedures are instigated. Cementum-specific proteins (CEMPs) could promote mitosis by increasing cytosolic Ca^2+^ levels and initiating protein kinase C (PKC) cascades, and ultimately foster the formation of new cementum and bone.^89,90^ Conversely, TGF-β1 signaling inhibits cementoblast differentiation while promotes fibroblast differentiation.^89,90^ Early experiments demonstrated that human PDLSCs transplanted with hydroxyapatite (HA) scaffolds into periodontal defects of mandibular molars in immunodeficient mice led to bone and PDL-like structure regeneration after eight weeks.^91,92^ Under similar conditions, collagen fibers formed in vivo connected newly generated cementum-like and bone-like structures, mimicking the physiological attachment of natural Sharpey fibers. These results highlight the potential of PDLSCs. However, further research is required to better understand the role of specific subpopulations in guiding targeted differentiation.

ABMSCs, SHEDs, and GMSCs also have stem cell characteristics and subpopulation heterogeneity (the known subpopulations of these cells are summarized in Supplementary Table 3). Although the present research progress slightly lags behind DPSCs and PDLSCs, we also have the possibility to establish a comprehensive understanding of their subpopulations from the existing achievements.

### (3) Alveolar bone mesenchymal stem cells (ABMSCs) / Jaw bone marrow mesenchymal stem cells (JBMMSCs)

Strictly speaking, alveolar bone is not technically classified as a component of dental tissues, however this structure is inseparable from teeth, as well as a part of the jaw, functioning as the “soil” for tooth development. Certain studies identified through scRNA-seq that only 1.74% of the cellular components of mouse mandibular alveolar bone were MSCs, which were further defined as four subpopulations: MSCs (*LepR*^*+*^), osteoblasts (*Bglap*^*+*^), endothelial cells (*Cdh5*^*+*^) and neural cells (*Plp1*^*+*^).^93^ To fully understand ABMSCs and their heterogeneity, we tried to expand our vision to the whole jaw structure and their inherent stem cells, namely jaw bone marrow mesenchymal stem cells (JBMMSCs). Unlike long bones such as femurs, which originate from the mesoderm and develop *via* endochondral osteogenesis, the jaw develops from neural crest cells in the ectoderm through intramembranous osteogenesis.^94^ Given that intrinsic variations and niche-specific effects may influence stem cell activity at different sites, an analysis of differentially expressed genes revealed notable differences in homeobox gene expression between mandibular and femoral BMMSCs in rats.^95^ Homeobox transcription factors, particularly those in the Hox superfamily—which regulate MSCs proliferation, maxillofacial development and tooth formation—were examined via scRNA-seq.^95^ BMMSCs from skeletal tissues generally exhibit Hox positivity, while JBMMSCs lack this characteristic.^95^ Gene ontology analysis further indicated that JBMMSCs showed elevated expression in pathways associated with osteoblast differentiation, SMAD signaling, cartilage development and glucose transmembrane transporter activity.^95^ Other related studies have observed a LepR^+^ subpopulation of BMSCs through lineage tracing, representing the main precursor of adult osteoblasts.^88^ The scRNA-seq data in one research revealed a newly identified osteolineage subpopulation that is characterized by the prominent expression of protocadherin FAT4 and transcription factor SOX6.^96^ Compared to long bone, the FAT4^+^ cells are especially enriched in alveolar bone, exhibiting a core transcriptional signature associated with osteogenesis, and may initiate a unique osteogenic differentiation trajectory of alveolar bone.^96^ These findings strongly indicated FAT4^+^ cells as distinct progenitor cells with osteogenic capacity of alveolar bone.

Due to variances in the developmental origins and the site specificity of the resident microenvironment, JBMMSCs have stronger self-renewal, osteogenic, and angiogenic potential. Compared with other stem cells like BMMSCs, the proliferative and differential functions of JBMMSCs decline less with aging, but the adipogenic and chondrogenic abilities are relatively weaker.^94,97^ These unique properties should be carefully considered for future therapeutic applications. For instance, several studies have proved that JBMMSCs possess superior properties, and would be ideal therapeutic stem cells for maxillofacial regeneration or other diseases.^97,98^ JBMMSCs are considered as the preferred resource for future therapeutic applications of craniofacial diseases (such as jaw tumors, jaw osteoporosis, and periodontal defects).^97,98^ Several studies have profiled its osteogenic differentiation potential and regulatory mechanism. Mechanical tension suppresses NF-κB in a non-inflammatory environment (NF-κB is thought to obstruct the osteogenesis of MSCs in vivo and in vitro by promoting the β-catenin degradation), and the expression and mineralization of osteoblast transcription factors in JBMMSCs increase and facilitate the osteogenesis.^99^ Additionally, miR-344d-3p has been found to stimulate osteogenic differentiation while simultaneously inhibit adipogenic differentiation of JBMMSCs.^100^ Cathepsin K (*CTSK*) gene knockout or suppressing can motivate alveolar bone regeneration by enhancing the regeneration of JBMMSCs via glycolysis.^101^ MiR-145 performs as an inhibitory factor in the osteogenic differentiation of JBMMSCs by directly targeting semaphorin3a (*SEMA3A*).^102^ However, osteogenic differentiation can be disrupted under certain conditions. For example, inflammation impairs the pluripotency of JBMMSCs, leading to diminished bone regeneration. These findings emphasize the importance of understanding the complex regulatory mechanisms and the microenvironmental factors influencing JBMMSCs to optimize their therapeutic potential in regenerative medicine.^99^

### (4) Stem cells from human exfoliated deciduous teeth (SHEDs)

The replacement of deciduous and permanent teeth is an orderly and dynamic process involving the resorption of deciduous tooth roots and the development and eruption of permanent teeth. Early research isolated a unique population of DMSCs called SHEDs from the dental pulp of exfoliated deciduous teeth.^103^ SHEDs exhibited strong potential for receptor cell-mediated bone formation in vivo and expressed neuronal and glial cell markers.^103^ Studies using surface antigen analysis of SHEDs demonstrated that over 99.5% of heterogeneous cells expressed CD105, CD73, and CD90 (markers for MSCs, hematopoietic stem cells and blood cells), while less than 3% expressed CD14, CD19, CD34, and CD45 (specific biomarkers for hematopoietic stem cells and blood cells).^104^

Flow cytometry analysis revealed that approximately 70.9% of SHEDs populations expressed CD146.^48,104,105^ Magnetic-activated cell sorting (MACS) technology has been employed to sort SHEDs.^48,104,105^ It reveals that CD146^+^ SHEDs share similarities with perivascular cells and exhibit higher osteogenic differentiation potential than unsorted SHEDs and CD146^−^ SHEDs.^48,104,105^ This suggests that the CD146^+^ subpopulation is more effective for osteogenic differentiation, while the CD146^−^ subpopulation has stronger adipogenic differentiation potential.^48,104,105^ Vascular endothelial growth factor (VEGF), acting as an autocrine factor, enhances the osteogenic capacity of SHEDs.^48,104,105^ CD146 serves as a coreceptor for VEGFR2, which regulates the neovascularization, and the VEGF-VEGFR2 interaction enhances pathways that promote β-FGF, BMP-2, RUNX2 and OSX expression to facilitate angiogenesis and bone regeneration.^48,104,105^ A study was conducted by transplanting heterogeneous populations of SHEDs, CD146^+^ and CD146^−^SHEDs, into bone defects generated in the skulls of immunodeficient mice.^106^ The outcomes demonstrated that bone regeneration was observed upon transplantation with CD146^+^ and heterogeneous populations of SHEDs, with significantly higher in CD146^+^ SHEDs.^106^ Meanwhile, bone regeneration in the CD146^−^ group was apparently lower than that in other transplant groups at 4 and 8 weeks.^106^ In addition, neural differentiation also varies among subpopulations: The mixed population and CD146^+^ subpopulation expresses glial cell markers, while the CD146^−^ subpopulation shows significantly increased expression of neural precursor and neuronal markers.^48^ Another study suggested a negative correlation between CD105 expression and osteogenic potential in SHEDs, with has-miR-1287 involving in the regulation of CD105 expression.^107^ SHEDs appear to display a unique *TLR* expression profile. Studies have found that in non-inflammatory microenvironments, SHEDs express *TLRs* (including *TLR1, TLR2, TLR3, TLR4, TLR6, TLR8, TLR9, and TLR10*), while in inflammatory microenvironments, they exhibit significant downregulation of *TLR7* at gene level and upregulation of *TLR8* at gene and protein levels.^108^ This characteristic indicates that SHEDs possess specific immune and regenerative abilities in oral tissue engineering.

### (5) Gingival mesenchymal stem cells (GMSCs)

Gingiva, the oral mucosa covering the neck and alveolar ridge of teeth, comprises epithelial layer and lamina propria, the latter of which develops from extracellular matrix formed by neural crest cell migration. Evidence suggests that fibroblasts in the free gingival lamina propria, derived from the inner layer of the dental follicle, contribute to the dentogingival fiber system and originate partly from PDL cells.^109^ Early studies have isolated and characterized GMSCs from human gingival tissues.^110^ These cells in vitro exhibit proliferation as plastic-adherent cells with fibroblast-like morphology, colony-forming abilities and multipotent differentiation potential.^110^ GMSCs exhibit weaker osteogenic potential compared to PDLSCs, but their unique characteristics lie in their high proliferative properties and the tendency to differentiate into neural cell lineages due to the neural crest origin.^101^ In accordance with previous researches, the majority of GMSCs (about 90%) originates from CNCCs, while only a small percentage (about 10%) derives from the mesoderm.^111,112^ CNCCs-derived GMSCs express neural crest-related genes such as *SOX9, TWIST1, SNAIL, MYC, ETS1, CRABP1, EPHA2*, and *ITGB1*, contributing to superior neurogenic and chondrogenic differentiation potential.^111,112^ This indicates that adult gingival tissues might act as a potential reservoir of neural crest-derived pluripotent stem cells.^111^ However, no significant differences are observed in osteogenic and adipogenic differentiation.^111,112^

However, investigations into the1. Subpopulations of GMSCs are still in their nascent stage. Recent evidence indicates that CD90^+^ GMSCs possess moderate osteogenic differentiation capacity, which provide a potential biomarker for identifying osteogenic subpopulations.^113^ Subpopulations with higher expression of *P53, SIRT1*, and *CDKN2A* exhibit effective DNA damage responses and reduced tumorigenicity with aging.^114^ Like MSCs from other tissues, GMSCs possess pluripotent differentiation potential and exhibit strong immunoregulatory effects on both innate and adaptive immune cells through the secretion of bioactive factors with immunosuppressive and anti-inflammatory properties.^111,115,116^ A recent study has demonstrated that B7-H1 plays a critical role in the immunosuppressive function of GMSCs via STAT3 signaling in a mouse model with collagen-induced arthritis.^117^ This highlights the potential for identifying new GMSCs subpopulations with enhanced immunosuppressive properties.^117^ These investigations clearly defined the precise characterization of GMSC subpopulations, thereby advancing their selective isolation for therapeutic applications.

## Molecular mechanisms underlying Dmscs heterogeneity

The observed heterogeneity among Dental Mesenchymal Stem Cells (DMSCs) goes beyond simply identifying subpopulations. It reveals that their functional diversity is closely governed by a conserved network of signaling pathways, which coordinately regulate both lineage specification and the maintenance of stemness.

Notably, the BMP-Smad pathway and the RUNX2/NELL-1 axis function as the core drivers of osteogenic and odontogenic differentiation. In DPSCs, the activation of BMP2-Smad1/5/8 signaling promotes odontoblast formation by upregulating key transcription factors such as DLX3, Sp7, and NELL-1.^65–67^ Conversely, excessive non-Smad signaling via P38/ERK can inhibit this process.^65–67^ Parallel to this, the Wnt/β-catenin pathway, often in concert with TGF-β1, balances osteogenic versus fibroblastic lineage commitment. This balance is mirrored in PDLSCs, where mechanical stimulation induces Wnt/β-catenin and TGF-β1 signaling to synergistically modulate the fibroblast-to-osteoblast transition, thereby promoting osteogenesis.^52,84,85,89^ Meanwhile, pathways such as Notch and PI3K/AKT, in cooperation with cytoskeletal proteins, sustain self-renewal, proliferation, and pro-angiogenic capacity in specific subpopulations. For instance, the NOTCH1–THY1 axis helps regulate cell cycle progression and maintains quiescence in DFSCs and DPSCs,^31,32,60,61^ while the PI3K/AKT cascade—highly active in PDGFRβ^+^ DPSC subpopulations—mediates angiogenic potential and supports tissue repair under hypoxic or inflammatory stress.^63^

Furthermore, these extracellular signals ultimately converge within the nucleus, where they are precisely decoded through transcriptional and epigenetic mechanisms. The cascade of transcription factors orchestrating hard tissue differentiation is activated, while epigenetic regulators such as EZH2 and miR-145 have been shown to fine-tune DMSC lineage commitment through chromatin remodeling and post-transcriptional repression of osteogenic genes, respectively.^86,102^ More profoundly, during differentiation, cells undergo chromatin remodeling to construct specific super-enhancers.^67,68^ This pre-established open chromatin state lays the groundwork for initiating the odontogenic-specific gene program, determining the direction and efficiency of cellular differentiation.

Overall, these interconnected pathways—BMP/Smad, Wnt/β-catenin, Notch, TGF-β, and PI3K/AKT—form a multi-dimensional regulatory network spanning from signaling to epigenetics, which constitutes the foundation of DMSC developmental and functional heterogeneity. Understanding how these mechanisms collectively govern tissue homeostasis maintenance and cellular differentiation will provide the molecular basis for targeted and precisely guided dental tissue regeneration.

## Conclusion

Research on DMSCs and their heterogeneity has significantly advanced, driven by the development of technologies such as scRNA-seq. The intricate structures of teeth and periodontal tissues are closely linked to the multi-functionality of TGPCs, DFSCs and SCAPs. The developmental heterogeneity exhibited by these cells dictates their differentiation into teeth and periodontal tissues development, deepening our understanding of the underlying mechanisms. After maturation, DMSCs (e.g., DPSCs, PDLSCs) stored in adult tissues retain notable heterogeneity. Distinct subpopulations with defined potentials (osteogenic, neurogenic) are stringently regulated by conserved signaling and epigenetics, facilitating tissue homeostasis and repair. This field still holds considerable potential for further exploration:

- Limitations: Some findings are constrained by technical artifacts, inconsistent experimental conditions, and the limited translatability of rodent models to human oral biology.
- Future prospects: Further research requires integrating multi-omics approaches, establishing standardized frameworks, performing rigorous in vivo validation, and overcoming clinical translation barriers to achieve tissue regeneration.

Persistent exploration into the heterogeneity of DMSCs will enhance our understanding of dental development mechanisms and provide a stronger foundation for clinical therapeutic applications.

## Literature search strategy

A systematic literature search was conducted across PubMed, Web of Science, and Scopus for studies published between January 2018 and September 2025, focusing on the heterogeneity of dental mesenchymal stem cells (DMSCs) and related cell populations. Using keyword combinations such as “(dental stem cells) AND (heterogeneity)” and specific DMSC subpopulations queries, approximately 860 records were initially retrieved. After duplicate removal, title/abstract screening, and full-text evaluation based on predefined inclusion/exclusion criteria—which required original data, investigation of cellular or molecular heterogeneity, and publication in peer-reviewed English journals—93 studies were ultimately included. The quality of studies was assessed considering experimental design, sample size, and model species, while potential technical limitations of methods like scRNA-seq were acknowledged in the synthesis of findings.

## Supplementary information

Supplementary Table 1

Supplementary Table 2

Supplementary Table 3
