# Supplementary material for: Research progress in heterogeneity of dental mesenchymal stem cells
Source: Int J Oral Sci. 2026 Apr 3;18:31. doi: 10.1038/s41368-026-00433-8 (PMC13049117; doi:10.1038/s41368-026-00433-8)
Supplement: Supplementary file 1 — Supplementary Table 1 [file 41368_2026_433_MOESM1_ESM.docx]

**Supplementary Table 1.** **Overview of DMSCs and DMSCs-Related Cells Classification and Characteristics**

| **Cell Types** | **Tissue Origin**  **& Development Stage** | | **Examples of Heterogeneity Performance (e.g. Typical Markers or Subpopulations)** |
| --- | --- | --- | --- |
| **Developing Tissues** | | |  |
| TGPCs | Tooth germ | Emerge during initial tooth germ formation, active in Bell Stage. | An assemblage of diverse primitive progenitors; different subpopulations follow distinct trajectories (e.g., *FGF3^+^* cells are odontoblast precursors). |
| DFSCs | Dental follicle | Emerge at Cap Stage with the formation of the dental follicle. | Distinct functional subpopulations:  • **PTHrP^+^ subpopulation**: Osteogenic and cementogenic potential.  • Subpopulation expressing neural progenitor markers. |
| SCAPs | Apical papilla of immature teeth | Emerge during the root development phase. | Remarkable functional heterogeneity:  • ***DLX5* expressing**: High odontogenic potential.  • ***DIO2* expressing**: High osteogenic potential.  • **CD146^+^ subpopulation**: Linked to angiogenesis.  • **STRO-1^+^ subpopulation**: Possesses neurogenic features. |
| **Adult Tissues** | | |  |
| DPSCs | Adult dental pulp (niche: vascular nerve bundle) | | High proliferation, multipotent, significant functional heterogeneity.  • **Osteogenic/Odontogenic Clusters** (expressing *DCN*, *COL1A1*)  • **Neurogenic Clusters** (expressing *S100A4*, *NEFM*)  • **Proliferative/Stemness Subpopulations** (high *SEPTIN*) |
| PDLSCs | PDL | | Strong repair/regeneration capacity, multipotent.  • **Fibroblastic-oriented** (expressing *ACTA2*, *CALD1*, *et al*.)  • **Osteogenic/Cementogenic** (expressing *RUNX2*, *POSTN*, *et al*.)  • **Progenitor/Precursor** (low collagen) |
| SHEDs | Pulp of Exfoliated Deciduous Teeth | | Very high proliferation, osteogenic & neurogenic potential.  • **CD146^+^ Subpopulation**: Strong **osteogenic/angiogenic** bias  • **CD146^-^ Subpopulation**: Strong **adiopgenic/neurogenic** bias |
| ABMSCs | Alveolar bone marrow | | Neural crest origin, strong osteogenic & angiogenic potential, age-resistant.  • ***LepR^+^* Subpopulation**: Main osteogenic precursor  • **FAT4^+^ Subpopulation**: Alveolar bone-specific osteoprogenitors |
| GMSCs | Gingival tissues | | Primarily neural crest-derived, high proliferation, strong immunomodulation, weak osteogenic.  • **Neural Crest-derived (Majority)**: Superior neuro/chondrogenic potential  • **CD90^+^ Subpopulation**: Possesses **osteogenic** potential |
