# Supplementary material for: Research progress in heterogeneity of dental mesenchymal stem cells
Source: Int J Oral Sci. 2026 Apr 3;18:31. doi: 10.1038/s41368-026-00433-8 (PMC13049117; doi:10.1038/s41368-026-00433-8)
Supplement: Supplementary file 2 — Supplementary Table 2 [file 41368_2026_433_MOESM2_ESM.docx]

**Supplementary Table 2. Developmental Heterogeneity of Temporarily Existing DMSCs**

| **Dental stem cell** | **Method used** | **Main findings** | **Ref.** |
| --- | --- | --- | --- |
| DFSCs | H & E staining; Real-Time PCR; Western blot analysis, etc. | ·Potential for differentiating into periodontal lineage cells.  Related signaling pathways: TGF-β signaling pathway, Hippo signaling pathway and Wnt signaling pathway through the crosstalk of BMP2, BMP4 and BAMBI.  Key regulatory pathway: RUNX2/NELL-1 axis. | (21)  (28)  (29)  (30) |
|  | Histological analysis; Immunocytochemistry analysis;  RT-PCR | ·Strong expression of NOTCH1 and NOTCH1 receptor.  Notch1 activation induced shortening of G1 phase and acceleration of S phase transition, potentially reducing their differentiation potential, whilst enhancing self-renewal and proliferation capacities. | (32) |
|  |  | ·Neural differentiation potential.  Approximately 70% of DFSCs expressed nestin and about 90% expressed β-III tubulin. | (32) |
|  | Histological analysis; Immunocyto-chemistry analysis;  ScRNA-seq | ·PTHrP^+^ DFSCs: Cementum cells may directly originate from PTHrP^+^ DFSCs.  Osteoblasts need a transitional cell population from the PTHrP^+^ DFSCs. | (33)  (34) |
| SCAPs | Proteomics analysis | ·High expression of CD44: Stimulates the differentiation of dental pulp and dentin cells. | (43) |
|  | RT-PCR; Real-Time PCR; Immunohisto-chemical staining; Western blot analysis, etc.  ·SCAPs-WIF1 cells possess stronger odontogenic potential. | WIF1 enhanced ALP activity and mineralization in vitro by activating the transcription factor OSX, thereby strengthening the dentin differentiation of SCAPs. | (45) |
|  | ScRNA-seq, GRN | ·*DLX5* expressing: stronger tooth formation ability.  ·Subpopulation with high expression of *DIO2* exhibits considerable osteogenic differentiation potential. | (46) |
|  | - | ·STRO-1^+^CD146^+^ subpopulation presented a higher proliferation rate and superior odontogenic differentiation potential.  CD146^+^ subpopulation may be linked to angiogenesis and endothelial cell activity.  STRO-1^+^ subpopulation contains neurogenic features. | (38)  (42) |
|  |  | ·CD24 may suggest the stemness state of SCAPs.  Once passaged to the 10^th^ generation, expression of CD24 will decrease to nearly 0%, meaning that cells have begun to transition into the osteoblast lineage. | (42) |
