# Supplementary material for: Research progress in heterogeneity of dental mesenchymal stem cells
Source: Int J Oral Sci. 2026 Apr 3;18:31. doi: 10.1038/s41368-026-00433-8 (PMC13049117; doi:10.1038/s41368-026-00433-8)
Supplement: Supplementary file 3 — Supplementary Table 3 [file 41368_2026_433_MOESM3_ESM.docx]

**Supplementary Table 3. ABMSCs, SHEDs, GMSCs and Their Heterogeneity**

| **Cell Populations** | **Method used** | **Main findings** | **Ref.** |
| --- | --- | --- | --- |
| ABMSCs/  JBMMSCs | ScRNA-seq | ·Mesenchymal stromal cells in mouse mandibular alveolar bone.  4 subpopulations: MSCs (*LepR^+^*), osteoblasts (*Bglap^+^*), endothelial cells (*Cdh5^+^*) and neural cells (*Plp1^+^*). | (93) |
|  | ScRNA-seq | ·BMMSCs from the maxillofacial region exhibited a lack of Hox superfamily positivity. | (95) |
|  | Lineage tracing | ·*LepR^+^* BMSCs subpopulation  Representing the main precursor of adult osteoblasts. | (88) |
|  | ScRNA-seq | ·FAT4^+^cells  Especially enriched in alveolar bone.  Exhibiting a core transcriptional signature associated with osteogenesis, and may initiate osteogenic differentiation trajectory of alveolar bone. | (96) |
| SHEDs | Flow cytometry analysis; MACS | ·CD146^+^ subpopulation  Sharing substantial similarity with peripheral cells and presenting higher bone differentiation potential.  ·CD146^-^ subpopulation  Stronger potential for adipogenic differentiation. | (48)  (104)  (105) |
|  |  | ·CD105 expression  Negatively related to osteogenic potential.  Has-miR-1287 is involved in its expressing regulation. | (107) |
|  | Flow cytometry analysis | ·TLR expression profile  In inflammatory microenvironments, it exhibits significant downregulation of TLR7 at gene level and upregulation of TLR8 at gene and protein levels. | (108) |
| GMSCs | Single Cell Cloning; Real-Time PCR | ·Cells with high CD90 expression  Moderate osteogenic potential. | (113) |
|  | Flow cytometry analysis; Real-Time PCR | ·Subpopulations with higher expression of *P53*, *SIRT1* and *CDKN2A.*  Presenting effective responses towards DNA damage and lower likelihood of age-related tumorigenicity. | (114) |
